# Clinical and Prognostic Relevance of Cycle Pattern Recognition in Bipolar Disorder: A Further Step Toward Personalised Treatment Pathways?

**DOI:** 10.1192/j.eurpsy.2026.10225

**Published:** 2026-07-31

**Authors:** A. E. Koukopoulos, D. Janiri, M. Pinto, G. Serra, L. De Chiara, A. M. D’Onofrio, G. Camardese, G. Sani

**Affiliations:** 1 Department of Life Science, Health, and Health Professions, Link Campus University; 2 Department of Neuroscience, Section of Psychiatry, Università Cattolica del Sacro Cuore; 3 Child and Adolescent Neuropsychiatry Unit, Bambino Gesù Children’s Hospital, Istituto di Ricovero e Cura a Carattere Scientifico (IRCCS); 4 Psychiatry, Struttura Residenziale Psichiatrica Samadi S.p.A., Rome, Italy

## Abstract

**Introduction:**

Bipolar disorder (BD) presents with heterogeneous longitudinal cycling patterns: manic-depressive (MD), depressive-manic (DM), and irregular (IRR). Although cycle type has long been considered a central feature, studies systematically examining its impact on clinical course remain limited.

**Objectives:**

To investigate associations between cycle type (MD, DM, IRR) and clinical-demographic features in a large, well-characterised sample of BD-I and BD-II patients, with focus on onset polarity, hospitalisations, suicidality, seasonality, predominant polarity, and diagnostic subtype.

**Methods:**

A total of 378 outpatients with DSM-5 BD-I/BD-II were assessed using life charts to classify cycle type. Clinical and demographic variables were collected via structured interviews and records. Group differences were tested with chi-square and ANOVA/Kruskal–Wallis; multivariate logistic regressions assessed independent associations.

**Results:**

Among 378 patients, 140 (37.0%) showed MD, 92 (24.3%) DM, and 146 (38.6%) IRR cycles. MD patients were more likely to present manic/hypomanic onset, manic predominant polarity, BD-I diagnosis, and higher hospitalisation rates. DM cycles strongly associated with depressive onset and depressive predominant polarity. Both MD and DM showed higher seasonality than IRR. BD-II was more frequent in DM and IRR. Suicidal ideation did not differ; suicide attempts were more frequent in IRR.

**Table 1.** Key clinical characteristics by cycle type Table 1. Long description.

Variable	MD (n=140)	DM (n=92)	IRR (n=146)
Manic onset %	52.9	4.3	14.4
Depressive onset %	17.8	92.4	65.7
Hospitalisation %	42.1	42.4	26.7
Seasonality %	32.9	36.9	4.8

**Table 2.** Predominant polarity distribution Table 2. Long description.

Predominant Polarity	MD %	DM %	IRR %
Manic	32.1	4.3	14.4
Depressive	9.3	39.1	33.6
Undetermined	58.6	56.5	52.0

**Table 3.** Diagnostic subtype Table 3. Long description.

Diagnosis	MD %	DM %	IRR %
BD-I	80.0	40.2	41.8
BD-II	20.0	59.8	58.2

**Conclusions:**

Cycle type represents a clinically meaningful longitudinal specifier in BD. Early recognition may inform prognosis and guide personalised preventive and therapeutic interventions. Systematic adoption of cycle pattern classification could enhance precision psychiatry in bipolar disorder.

**Disclosure of Interest:**

None Declared

## Long descriptions

### Table 1. Long description

The table consists of four columns: Variable, M D (n=140), D M (n=92), and I R R (n=146).

* Manic onset percentage: M D is 52.9, D M is 4.3, and I R R is 14.4.

* Depressive onset percentage: M D is 17.8, D M is 92.4, and I R R is 65.7.

* Hospitalisation percentage: M D is 42.1, D M is 42.4, and I R R is 26.7.

* Seasonality percentage: M D is 32.9, D M is 36.9, and I R R is 4.8.

Navigate back to Table 1..

### Table 2. Long description

The table consists of four columns and four rows including the header.

Column headers from left to right are: Predominant Polarity, M D %, D M %, and I R R %.

Row 1: Manic polarity shows 32.1% for M D, 4.3% for D M, and 14.4% for I R R.

Row 2: Depressive polarity shows 9.3% for M D, 39.1% for D M, and 33.6% for I R R.

Row 3: Undetermined polarity shows 58.6% for M D, 56.5% for D M, and 52.0% for I R R.

Navigate back to Table 2..

### Table 3. Long description

The table consists of four columns and three rows.

Column headers from left to right are: Diagnosis, M D percentage, D M percentage, and I R R percentage.

Row 1: B D dash I. M D is 80.0 percent. D M is 40.2 percent. I R R is 41.8 percent.

Row 2: B D dash I I. M D is 20.0 percent. D M is 59.8 percent. I R R is 58.2 percent.

Navigate back to Table 3..

